# Supplementary material for: A Content Analysis of Persuasive Appeals Used in Media Campaigns to Encourage and Discourage Sugary Beverages and Water in the United States
Source: Int J Environ Res Public Health. 2023 Jul 13;20(14):6359. doi: 10.3390/ijerph20146359 (PMC10379826; doi:10.3390/ijerph20146359)
Supplement: Supplementary file 1 [file ijerph-20-06359-s001.zip › ijerph-2321332-supplementary.pdf]

Kraak VI, Holz Ivory A, Woods CL, Whitlow AR, Leary N. Kraak VI, Holz Ivory A, Woods C, Woods A, Leary N. A content analysis of persuasive appeals used in media campaigns to encourage and discourage sugary beverages and water in the United States. *Int J Environ Res Public Health.* 2023.

**S File 1**	Descriptive Information
	Table 1: Codebook for the research questions (RQ) 1 and 2 analysis
	Table 2: Codebook for the RQ 3 analysis
	References
**S File 2**	Comprehensive list of U.S. media campaigns (n=280), organized chronologically by the media campaign typology, used for the RQ1 and RQ2 analysis and unique media campaigns (n=60) used for the RQ3 analysis
**S File 3**	Fair use evaluation for the media campaign images used in Figure 2 to provide beverage campaigns used to promote or discourage sugary beverages and encourage healthy hydration

Supplemental File 1

1. Descriptive Information

2. Supplemental Table 1: Codebook for RQ1 and RQ2 analysis

3. Supplemental Table 2: Codebook for RQ3 analysis

4. References

1. Descriptive Information

**Variable Name**	**Description**
**caseid**	Label each individual slogan as an individual case.
**slogan**	The title of the campaign slogan, including words, grammar and punctuation.
**brand_organization**	The brand or organization responsible for the slogan.

**campaign_category**<br><br>1 = corporate advertising, marketing or entertainment campaigns<br>2 = corporate social responsibility, public relations, or cause marketing campaigns<br>3 = social marketing campaigns<br>4 = public information, awareness, education or health promotion campaigns<br>5 = media advocacy or countermarketing campaigns<br>6 = political or public policy campaigns	The advertisement's affiliated U.S. media campaign typology, as assigned by Kraak et al. (2022)
**year**	The date the campaign was launched.

2. Supplemental Table 1: Codebook for RQ1 and RQ2 analysis

APPEALS

**Variable Name**	**Description**
**rational**<br>0 = No<br>1 = Yes	*The rational variable name denotes a rational appeal.*<br>The campaign tagline or slogan aims to persuade audiences through rational thought processes and logical information to appeal to one's reason. The tagline or slogan provides the audience with fact-based and verifiable information about the product or brand, persuades by means of logical information and presentation of facts and data, or by raising awareness by means of presenting information (Casias & Pereira, 2021).<br><br>Only information within the text of the campaign slogan may be used to make this determination; images, background information about the campaign will not be used.

***Casais and Pereira (2021) use the term categories of analysis for the appeals that are listed alphabetically and defined below.**

Rational appeals include: comparative, factual, scarcity and solutions (n=4).
Emotional appeals include: positive emotional appeals (i.e., humor, pleasure, pride, relaxed, sexual and social) (n=6); negative emotional appeals (i.e., anger, disgust, fear, guilt and worry) (n=5); and co-active emotional appeals (n=1).

**comparative**<br>0 = No<br>1 = Yes	"Either directly or indirectly naming competitors in an advertisement and comparing one or more specific attributes (Hornick et al., 2016, p. 198).

factual
0 = No
1 = Yes

Something that is presented as having happened or existing, especially something for which proof exists, or about which there is information (Cambridge Dictionary). To think about something in a logical, sensible way (Oxford English Dictionary) grounded in evidence or factual information. Thinking in which logical processes of an inductive or deductive character are used to draw conclusions from facts or premises or both. (APA Dictionary of Psychology).

The tagline or slogan may include product-related information, including specific references to calories, price, process, or ingredients.

scarcity
0 = No
1 = Yes

The tagline or slogan seeks to "induce product purchase by triggering consumer inferences about product quality and/or product desirability," as well as consumers' desire for uniqueness or psychological reactance motivation.

solutions
0 = No
1 = Yes

A proposed actionable strategy or means to help an individual solve a problem or deal with a situation at individual, local, or global levels. Presented solutions are concrete and not just abstract or motivational.

EMOTIONAL APPEALS

emotional
0 = No
1 = Yes

The emotional variable name denotes an emotional appeal strategy.
The campaign tagline or slogan aims to persuade the target audience to prefer, buy or consume a product or a specific brand; or discontinue a behavior using affective stimuli that influences their emotions. The tagline or slogan may use positive appeals (i.e., arousal, enthusiasm or pleasure); negative appeals (i.e., anger, disgust or fear); or both (Casais & Pereira, 2021).

Only information within the text of the campaign slogan may be used to make this determination; images, background information about the campaign will not be used.

Viewers can perceive emotional appeals as either positive (pleasant), negative (unpleasant), or co-active (a combination of both positive and negative). Emotional appeals will be divided into three domains (positive, negative, coactive) based on valence or tone and operationalized below.

POSITIVE APPEAL

positive
0 = No
1 = Yes

The positive variable name denotes a positive emotional appeal.
The campaign tagline or slogan offers an incentive or benefits related to one's behavior to buy or consume a product, adopt a specific brand, or discontinue a behavior.

The tagline or slogan may include strategies such as humor, happiness, hope, pride, or love. Examples of words potentially used in this category include terms pertaining to life, health, smile, love, respect, protect, good, responsibility, save, gains, efficacy. The slogan may include rhetoric involving humor, empathy, motivating, and advising (Casais & Proenca, 2021).

Only information within the text of the campaign slogan may be used to make this determination; images, background information about the campaign will not be used.

Casais and Pereira (2021) list these as types of positive appeals or categories of analysis.

Types of emotional appeals (categories of analysis) are presented alphabetically and defined below. Emotional appeals include: positive emotional appeals (i.e., humor, pleasure, pride, relaxed, sexual and social) (n=6); negative emotional appeals (i.e., anger, disgust, fear, guilt and worry) (n=5); and co-active emotional appeal (n=1).

humor
0 = No
1 = Yes

The capacity to perceive or express the amusing aspects of a situation (APA Dictionary of Psychology).

relaxed
0 = No
1 = Yes

The tagline or slogan implies "warmth, a highly positive emotion that people enjoy in their relationships with family or friends" (Mensa et al., 2020, p. 4) and may include a "suggestion that this product will help you unwind" or provide comfort (Cheng & Schweitzer, 1996).

This category includes relief.

sexual
0 = No
1 = Yes

References to a "sexual encounter" or inclusion of "references in the text of the ad such as naughty, discreet, tempting." It may also reference "a romantic setting" or words like "passion" that are "connected to sex appeal or romance."

This category includes romantic.

social
0 = No
1 = Yes

The tagline or slogan may reflect individuals enjoying active lifestyles, parties or other occasions that suggest shared, social experiences.

"The use of a product is claimed to be able to elevate the position or rank of the user in the eyes of others." Product use conveys feelings of prestige and trendsetting (Cheng & Schweitzer, 1996, p. 30).

It may indicate wealth or affluence through "general references to success in terms of high social or economic status." It may also attempt to associate the product with some form of social success.

This category includes affluence, acceptance, belonging, success and status.

NEGATIVE APPEAL

**negative**<br>0 = No<br>1 = Yes	*The negative variable name denotes a negative emotional appeal.*<br>The campaign tagline or slogan aims to evoke discomfort or emotional imbalance toward a product or brand. The tagline or slogan may elicit anger, disgust, fear, guilt, shame, worry or threat in the target audience to disincentivize buying or using a product or brand (Casais & Pereira, 2021; Casais & Proença, 2021).<br><br>This category includes consequences, danger, death, disability, disease, fatality, fear, guilt, loss, shame or threat.<br><br>Only information within the text of the campaign slogan may be used to make this determination; images, background information about the campaign will not be used.

Types of negative emotional appeals (n=5), also called categories of analysis,* are listed alphabetically and defined below.

***Casais and Pereira (2021) list these as types of negative appeals or categories of analysis.**

**Anger**<br>0 = No<br>1 = Yes	Definition: A strong feeling of displeasure, dissatisfaction, or annoyance, generally combined with antagonism or hostility towards a particular cause or object (Oxford English Dictionary). An emotion characterized by tension and hostility arising from frustration, real or imagined injury by another, or perceived injustice. It can manifest itself in behaviors designed to remove the object of the anger or behaviors designed merely to express the emotion (APA Dictionary of Psychology).
**Disgust**<br>0 = No<br>1 = Yes	Definition: A feeling of revulsion or strong disapproval aroused by something unpleasant or offensive.<br><br>This category includes revolt, repulse, nauseate, unappetizing and unpalatable.
**Fear**<br>0 = No<br>1 = Yes	Definition: A basic, intense emotion aroused by the detection of imminent threat or consequences of one's choices or actions, involving an immediate alarm reaction or longer-term effect on behaviors that mobilizes the organism by triggering a set of physiological changes (APA Dictionary of Psychology).

**Guilt**<br>0 = No<br>1 = Yes	Definition: A failure of duty, delinquency; offense, crime, sin (Oxford English Dictionary). May also refer to a self-conscious emotion characterized by a painful appraisal of having done (or thought) something that is wrong and often by a readiness to take action designed to undo or mitigate this wrong (APA Dictionary of Psychology). This code may include guilt based on a previous action or may reflect guilt on an anticipatory basis. an emotional state that involves feelings of remorse, self-blame and self-punishment experienced after committing an action or contemplating future violation of internalized or socially acceptable standards of behavior.<br><br>An emotional state that involves feelings of remorse, self-blame and self-punishment experienced after committing an action or contemplating future violation of internalized or socially acceptable standards of behavior. There are three types of guilt: reactive, anticipatory and existential guilt. There are three types of guilt: reactive, anticipatory and existential guilt (Huhmann & Brotherton, 1997).<br><br>This category includes remorse, regret, and sorrow.
**Worry**<br>0 = No<br>1 = Yes	Definition: A state of mental distress or agitation due to concern about an impending or anticipated event, threat, or danger (APA Dictionary of Psychology).

COACTIVE APPEAL

**coactive**<br>0 = No<br>1 = Yes	*The coactive variable name denotes a coactive/mixed emotional appeal.*<br>The campaign slogan contains both positive and negative emotional appeals or a mixed state of emotions. Thus, they induced both positive and negative feelings at the same time. Only information within the text of the campaign slogan may be used to make this determination; images, background information about the campaign will not be used.

3. Supplemental Table 2: Codebook for RQ3 analysis

VARIABLE NAME	DESCRIPTION
**caseid**	Label each individual advertisement as an individual case.
**Image**	The advertisement image.
**Brand_organization**	The brand or organization responsible for the slogan.

Campaign_category
1 = corporate advertising, marketing or entertainment campaigns
2 = corporate social responsibility, public relations, or cause marketing campaigns
3 = social marketing campaigns
4 = public information, awareness, education or health promotion campaigns
5 = media advocacy or countermarketing campaigns
6 = political or public policy campaigns

The advertisement's affiliated U.S. media campaign typology, as assigned by Kraak et al. (2022).

People (5 variables)
0 = No
1 = Yes

The 'people'' variable name denotes the inclusion of people in the advertisement. People may include celebrities and other public figures, historical figures, or models. This variable includes images that feature an entire body/face or those which feature only a small portion of the body, such as hands or a partial face. The advertisement's visual elements may be used to make this determination; taglines, slogans, background information about the campaign will not be used.

Descriptions of people (categories of analysis) are listed below:
Note: Turner et al. (2020) list these as types of demographic elements or categories of analysis.

Biological_sex
0 = No individuals pictured
1 = Unclear
2 = Both male and female
3 = Male
4 = Female

The 'biological or sex' variable name denotes perceived biological sex of the person(s) pictured in the advertisement.

age
0 = No individuals pictured
1 = Unclear
2 = Multiple ages pictured
3 = Elementary school-aged children
4 = Teens/adolescents (up to age 18)
5 = Adults (18 and older)

The 'age' variable name denotes the perceived age of the person(s) pictured in the advertisement.

race_ethnicity
0 = No individuals pictured
1 = Unclear

The 'race or ethnicity' variable name denotes a "crude estimate" of the perceived race and ethnicity of people pictured in the advertisement.

2 = Multiple races or ethnicities pictured
3 = White
4 = Black
5 = Latinx or Hispanic
6 = Native American or Pacific Islander
7 = Asian

Perceived advertisement narratives and visual elements (categories of analysis) are listed below Casais & Proença (2015, 2021) and Alkazemi & Van Stee (2020) define these as perceived advertisement narratives, visual elements, or categories of analysis.

**emotion**<br>0 = unclear<br>1 = positive<br>2 = negative<br>3 = coactive	The 'emotion' variable name reflects the perceived emotional state of the person(s) in the advertisement. (Casais & Proença, 2021).<br><br>'not applicable' emotion indicates the advertisement does not contain people or the perceived emotional state of the person(s) is not distinguishable.<br>A 'positive' emotion state shows "motivating/confident people, social models, testimonial, public figures" (Casais & Proença, 2021). Facial expressions and body language reflect those that "are typically associated with positive emotions," including "smiling, laughing, hugging, etc." (Alkazemi & Van Stee, 2020).<br>A 'negative' emotion shows "frightened/worried people" (Casais & Proença, 2021). Facial expressions and body language reflect those that "are typically associated with negative emotions," such as "frowning, crying, etc." (Alkazemi & Van Stee, 2020).
**content**<br>0 = unclear<br>1 = positive<br>2 = negative<br>3 = coactive	The 'content' variable name reflects the "story content of the advertisements," including text and graphics (Casais & Proença, 2021).<br><br>'Unclear' story content indicates the advertisement does not contain story content or the valence (tone) of the story content is unclear or not distinguishable.<br>'Positive' story content shows the "benefits" of behaviors<br>'Negative' story content shows "negative or unwanted consequences" of behaviors<br>'Coactive' story content shows both the benefits and negative/unwanted consequences of a behavior
**color**<br>0 = multiple or no dominant color<br>1 = blue	The 'color' variable reflects the dominant color of the advertisement, meaning "the color that occupied the largest area of the ad," excluding black or white (Madden et al., 2000).

2 = brown<br>3 = gray<br>4 = green<br>5 = orange<br>6 = red<br>7 = violet<br>8 = yellow<br>9 = black<br>10 = traffic light color scheme (red, amber yellow, green)<br>11 = patriotic color scheme (red, white, and blue)	'No dominant color' indicates the advertisement did not feature a single dominant color.<br><br>The listed color options (blue, brown, gray, green, orange, red, violet, yellow) have "been established as standard hues that are readily observable in content analysis" (Madden et al., 2000).
**Color_qualitative**	Qualitatively note observances about the colors used in the advertisement. May include notes about emotions evoked by color scheme.
**product**<br>0 = absent<br>1 = present	The 'product image' variable name reflects the visual depiction of a product. This category excludes company logos (Alkazemi & Van Stee, 2020). This includes content, color, image and brand.<br>'Present' indicates the advertisement contains a visual depiction of the product<br>'Absent' indicates the advertisement does not contain a visual depiction of the product.

4. References

18. Casais, B.; Pereira, A.C. The prevalence of emotional and rational tone in social advertising appeals. *RAUSP Manag. J.* **2021,** *56*, 282–294. https://doi.org/10.1108/RAUSP-08-2020-0187.

28. Kraak, V.I.; Consavage Stanley, K.; Harrigan, P.B.; Zhou, M. How have media campaigns been used to promote and discourage healthy and unhealthy beverages in the United States? A systematic scoping review to inform future research to reduce sugary beverage health risks to Americans. *Obesity Rev.* **2022,** *23*, e13425. https://doi.org/10.1111/obr.13425.

29. Casais, B., Proença, J.F. The use of positive and negative appeals in social advertising: a content analysis of television ads for preventing HIV/AIDS. *Int. Rev. Public Nonprofit Mark.* **2021,** *19,* 623–647. https://doi.org/10.1007/s12208-021-00318-y.

48. Mensa, M.; Vargas-Bianchi, L. Nurtured and sorrowful: Positive and negative emotional appeals in COVID-19 themed brand communications. *SocArXiv* **2020**, https://doi.org/10.31235/osf.io/68ukd.

52. Alkazemi, M.F.; Van Stee, S.K. Electronic direct-to-consumer advertising of pharmaceuticals: An assessment of textual and visual content of websites. *Health Ed. Res.* **2020**, *35*, 134–151. https://doi.org/10.1093/her/cyaa004.

54. Turner, M.M.; Ford, L.; Somerville, V.; Javellana, D.; Day, K.R.; Lapinski, M.K. The use of stigmatizing messaging in anti-obesity communications campaigns: Quantification of obesity and stigmatization. *Commun. Rep.* **2020,** *33*, 107–120. https://doi.org/ 0.1080/08934215.2020.1793375.

59. Hornick, J.; Ofir, C.; Rachamim, M. Qualitative evaluation of persuasive appeals using comparative meta-analysis. *The Comm. Rev.* **2016,** *19*, 192–222. https://doi.org/10.1080/10714421.2016.1195204.

63. Madden, T.J.; Hewett, K.; Roth, M.S. Managing images in different cultures: A cross-national study of color meanings and preferences. *J. Int. Mark.* **2000**, *8*, 90–107. https://doi.org/10.1509/jimk.8.4.90.19795.

68. American Psychological Association. APA Dictionary of Psychology. 2002. Available online: https://dictionary.apa.org/ (accessed on 6 March 2023).

69. Lustig, R.H. *The Hacking of the American Mind: The Science behind the Corporate Takeover of Our Bodies and Brains*. Avery Random House LLC: New York, NY, USA, 2017. Available online: https://robertlustig.com/hacking/ (accessed on 6 March 2023).

71. Cheng, H.; Schweitzer, J.C. Cultural values reflected in Chinese and U.S. television commercials. *J. Advert. Res.* **1996**, *36*, 27–45.

76. Huhmann, B.A.; Brotherton, T.P. A content analysis of guilt appeals in popular magazine advertisements. *J. Adv.* **1997**, *26*, 35–46. https://doi.org/10.1080/00913367.1997.10673521.
